# Supplementary figures and images for: CCRK/CDK20 regulates ciliary retrograde protein trafficking via interacting with BROMI/TBC1D32
Source: PLoS One. 2021 Oct 8;16(10):e0258497. doi: 10.1371/journal.pone.0258497 (PMC8500422; doi:10.1371/journal.pone.0258497)

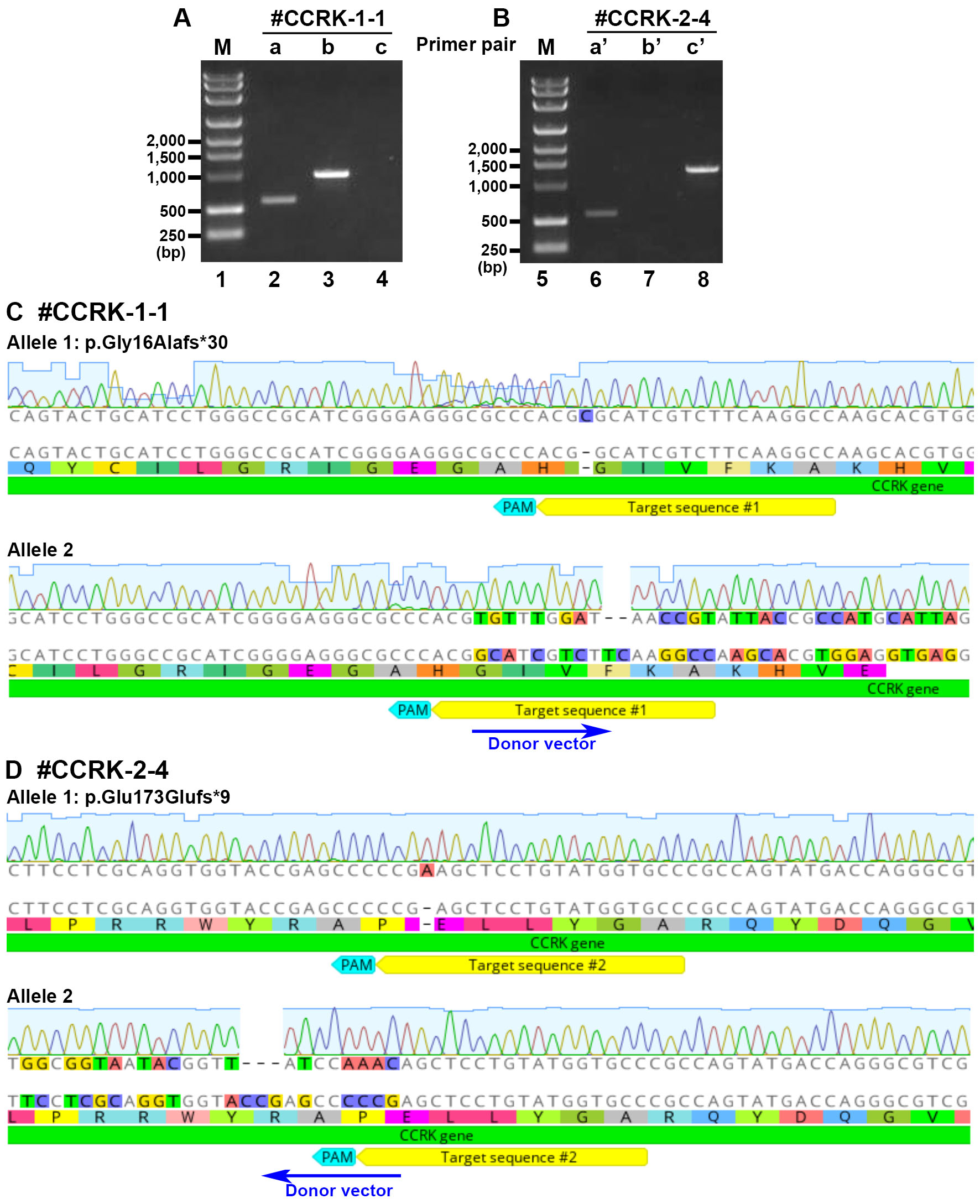

Supplement: S1 Fig — (A, B) Genomic DNAs were extracted from the CCRK-KO cell lines #CCRK-1-1 (A) and #CCRK-2-4 (B), which were established using a donor knockin vector containing distinct target sequences, and subjected to PCR analysis using the indicated primer sets (see S2 Table) to detect alleles with a small indel or no insertion (a, a’), or with forward (b, b’) or reverse (c, c’) integration of the donor knockin vector. M, molecular weight markers (PSU1 + ladder). (C, D) Alignments of allele sequences of the #CCRK-1-1 (C) and #CCRK2-4 (D) cell lines determined by sequencing of the PCR products shown in (A) and (B). Positions of the target sequence and protospacer adjacent motif (PAM) sequence, and insertion sites and directions of the donor knockin vector are indicated. (JPG) [file pone.0258497.s001.jpg]
