# Supplementary material for: CCRK/CDK20 regulates ciliary retrograde protein trafficking via interacting with BROMI/TBC1D32
Source: PLoS One. 2021 Oct 8;16(10):e0258497. doi: 10.1371/journal.pone.0258497 (PMC8500422; doi:10.1371/journal.pone.0258497)
Supplement: S2 Fig — (PPTX) [file pone.0258497.s002.pptx]

## Slide 1
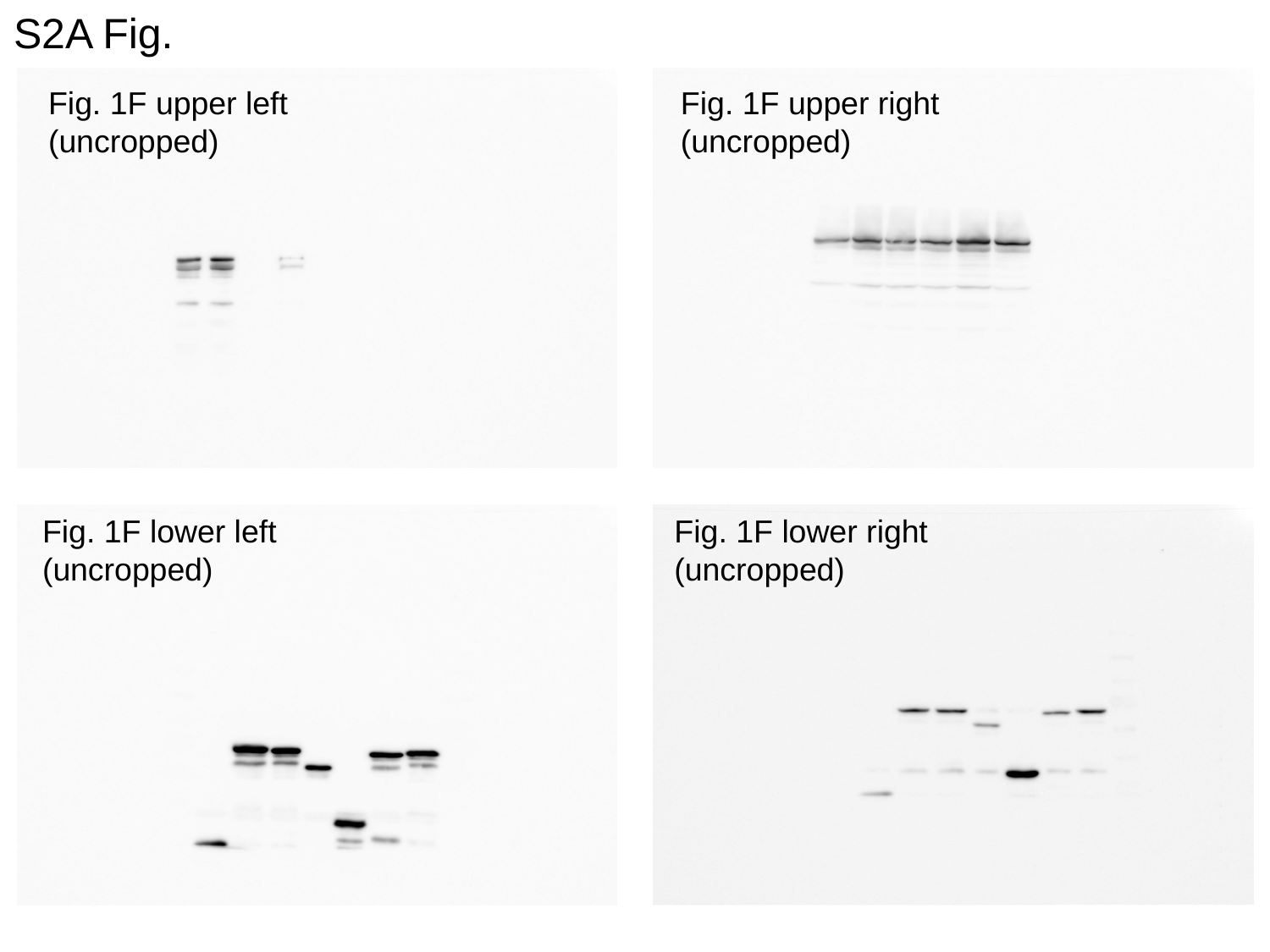

S2A Fig.
Fig. 1F upper left
(uncropped)
Fig. 1F upper right
(uncropped)
Fig. 1F lower left
(uncropped)
Fig. 1F lower right
(uncropped)

## Slide 2
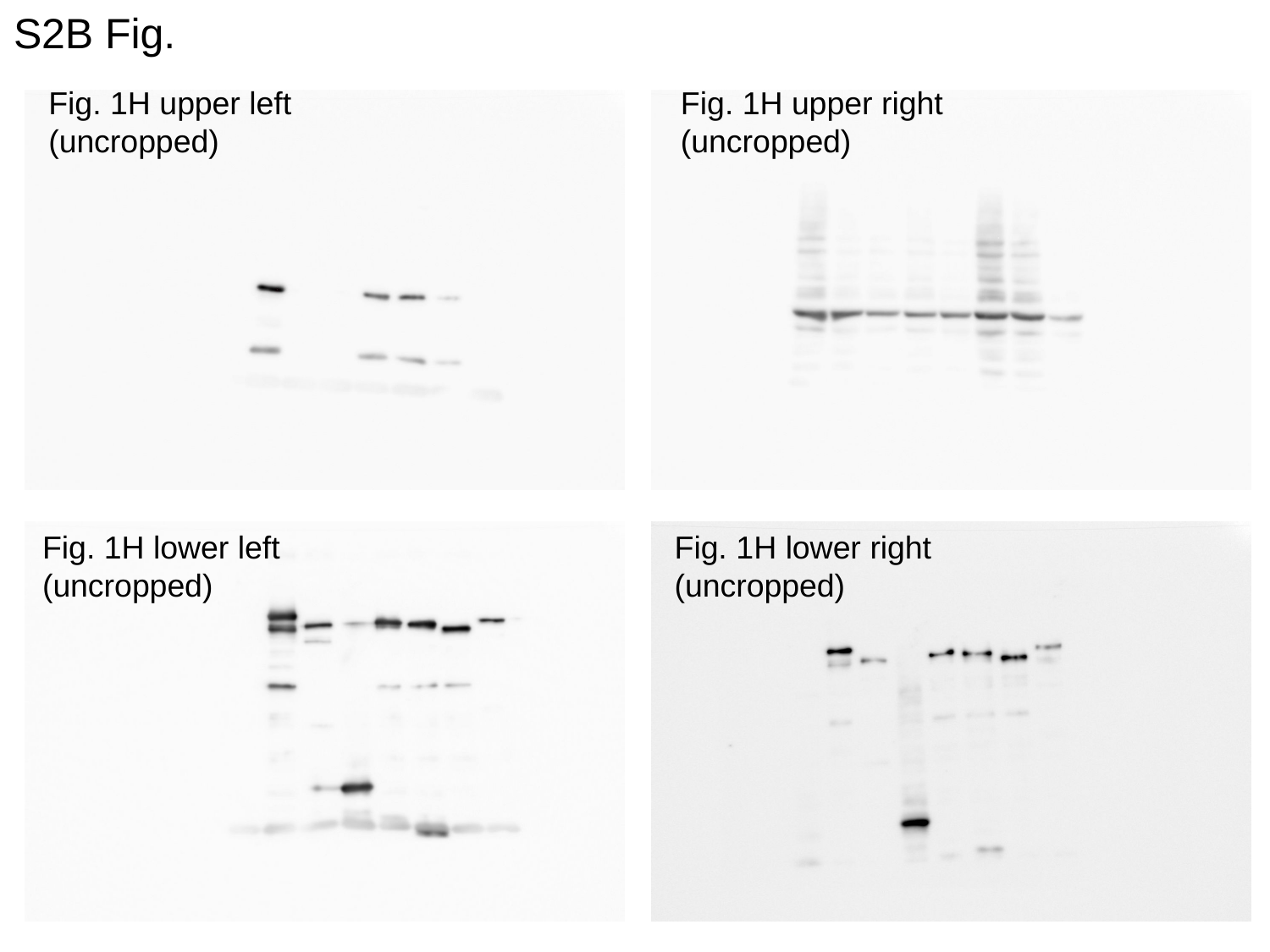

S2B Fig.
Fig. 1H upper left
(uncropped)
Fig. 1H upper right
(uncropped)
Fig. 1H lower left
(uncropped)
Fig. 1H lower right
(uncropped)

## Slide 3
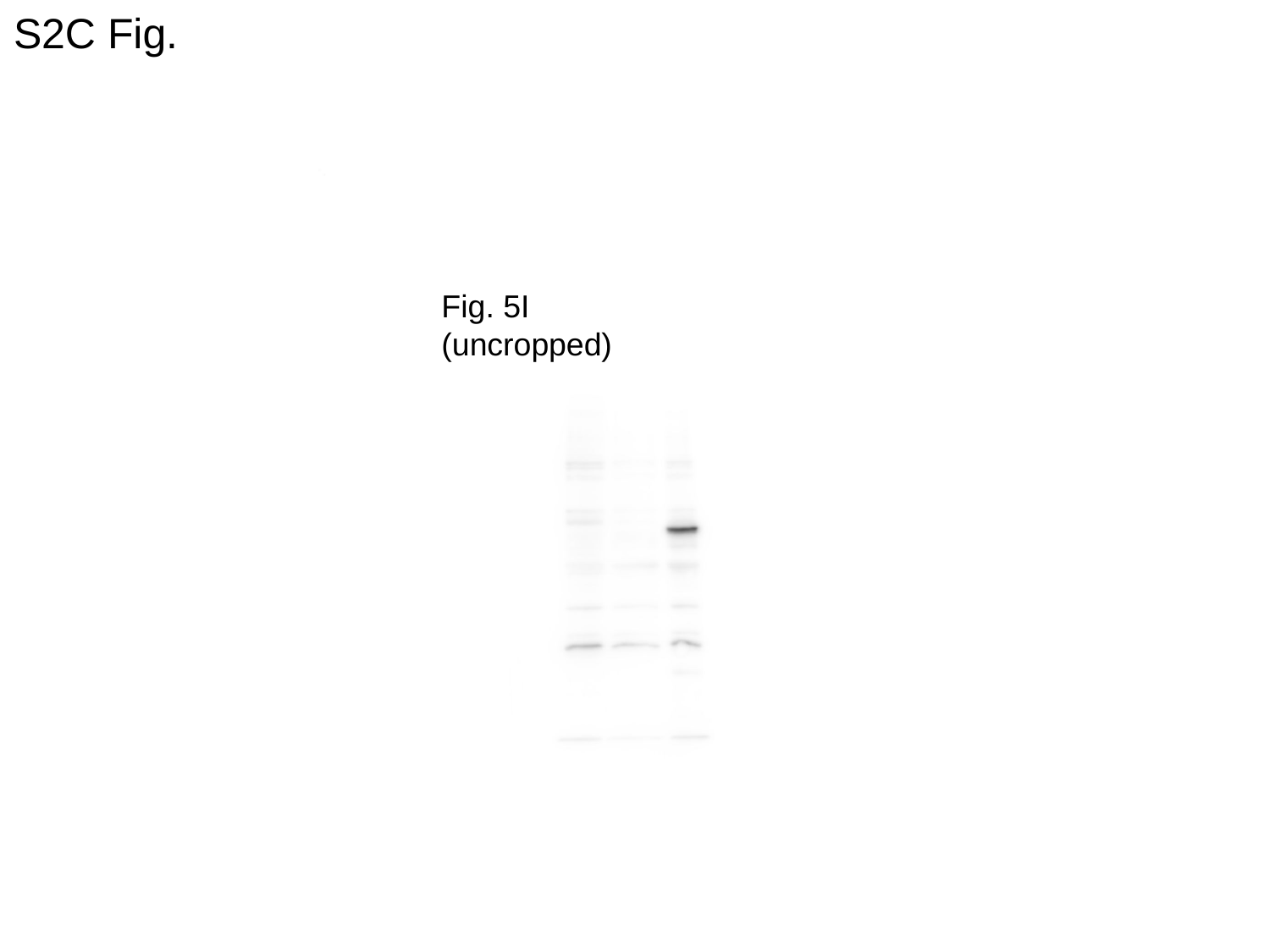

S2C Fig.
Fig. 5I
(uncropped)
