# Supplementary material for: CCRK/CDK20 regulates ciliary retrograde protein trafficking via interacting with BROMI/TBC1D32
Source: PLoS One. 2021 Oct 8;16(10):e0258497. doi: 10.1371/journal.pone.0258497 (PMC8500422; doi:10.1371/journal.pone.0258497)
Supplement: S1 Table — (PDF) [file pone.0258497.s003.pdf]

S1 Table. Plasmids used in this study

Vector	Insert	Reference
pCAG2-EGFP-N1	CCRK	This study
pCAG2-EGFP-N1	CCRK(K33R)	This study
pCAG2-EGFP-N1	CCRK(1–289)	This study
pCAG2-EGFP-N1	CCRK(1–330)	This study
pCAG2-EGFP-N1	CCRK(290–346)	This study
pCAG2-EGFP-N1	BROMI	This study
pCAG2-EGFP-N1	BROMI(1–1,101)	This study
pCAG2-EGFP-N1	BROMI(1,102–1,298)	This study
pCAG2-EGFP-N1	BROMI(157–1,298)	This study
pCAG2-EGFP-N1	BROMI(182–1,298)	This study
pCAG2-EGFP-N1	BROMI(243–1,298)	This study
pCAG2-EGFP-N1	BROMI(1–1,190)	This study
pCAG2-mCherry-N1	CCRK	This study
pCAG2-mCherry-N1	BROMI	This study
pRRLsinPPT-EGFP-N-IRES-Blast	CCRK	This study
pRRLsinPPT-EGFP-N-IRES-Blast	CCRK(K33R)	This study
pRRLsinPPT-EGFP-N-IRES-Blast	CCRK(1–289)	This study
pRRLsinPPT-EGFP-N-IRES-Blast	CCRK(1–330)	This study
pRRLsinPPT-EGFP-N-IRES-Zeo	ARL13B(ΔGD; 1–19, 190–428 aa)	Ref. 30
pRRLsinPPT-EGFP-N-IRES-Zeo	KAP3	This study
pRRLsinPPT-EGFP-C-IRES-Blast	DYNC2LI1	Ref. 71
pRRLsinPPT-uORF-mCherry-C-IRES-Blast	ICK	Ref. 30
pRRLsinPPT-uORF-mCherry-C-IRES-Blast	ICK(T157A)	Ref. 30
pRRLsinPPT-uORF-mCherry-C-IRES-Blast	ICK(T157E)	Ref. 30
pDonor-tBFP-NLS-Neo (Universal)	–	Ref. 61
pHiFiCas9-2×sgRNA	–	This study
pGEX-6P1	Anti-GFP Nb	Ref. 59

All cDNA inserts except for that of anti-GFP Nb are of human origin.
