# Supplementary material for: CCRK/CDK20 regulates ciliary retrograde protein trafficking via interacting with BROMI/TBC1D32
Source: PLoS One. 2021 Oct 8;16(10):e0258497. doi: 10.1371/journal.pone.0258497 (PMC8500422; doi:10.1371/journal.pone.0258497)
Supplement: S2 Table — (PDF) [file pone.0258497.s004.pdf]

S2 Table. Antibodies used in this study

Antibody	Manufacturer or provider	Clone/catalog number or reference	Dilution (purpose)
Polyclonal rabbit anti-IFT88	Proteintech	13967-1-AP	1:500 (IF)
Polyclonal rabbit anti-IFT140	Proteintech	17460-1-AP	1:500 (IF)
Polyclonal rabbit anti-GPR161	Proteintech	13398-1-AP	1:200 (IF)
Polyclonal rabbit anti-ARL13B	Proteintech	17711-1-AP	1:1,000 (IF)
Monoclonal mouse anti-ARL13B	Abcam	N295B/66	1:500 (IF)
Monoclonal mouse anti-SMO	Santa Cruz	sc-166685	1:100 (IF)
Monoclonal mouse anti-FOP	Abnova	2B1	1:5,000 (IF)
Monoclonal mouse anti-Ac-α-tubulin	Sigma-Aldrich	6-11B-1	1:1,000 (IF)
Monoclonal mouse anti-γ-tubulin	Sigma-Aldrich	GTU88	1:1,000 (IF)
Monoclonal mouse anti-RFP	MBL	3G5	1:1,000 (IF)
Monoclonal mouse anti-GFP	Proteintech	66002-1-Ig	1:5,000 (IF), 1:10,000 (IB)
Polyclonal rabbit anti-mCherry	Proteintech	26765-1-AP	1:10,000 (IB)
AlexaFluor-conjugated secondary	Molecular Probes	A11034, A21131, A21147, A21137, A21242, A21240	1:1,000 (IF)
Peroxidase-conjugated secondary	Jackson ImmunoResearch	115-035-166, 111-035-144	1:3,000 (IB)

IF, immunofluorescence; IB, immunoblotting
