# Supplementary material for: CCRK/CDK20 regulates ciliary retrograde protein trafficking via interacting with BROMI/TBC1D32
Source: PLoS One. 2021 Oct 8;16(10):e0258497. doi: 10.1371/journal.pone.0258497 (PMC8500422; doi:10.1371/journal.pone.0258497)
Supplement: S3 Table — (PDF) [file pone.0258497.s005.pdf]

**S3 Table: Oligo DNAs used in this study**

| Name | Sequence |
|---|---|
| pTagBFP-N-RV2 | 5'-CGTAGAGGAAGCTAGTAGCCAGG-3' |
| CCRK-genome#1-FW | 5'-GGTGGAGGAGAAGTGGAGTTTG-3' |
| CCRK-genome#1-RV | 5'-CTCCTGCAGAGCCTTAATCTCC-3' |
| CCRK-genome#2-FW | 5'-AGTCTTCTCCATGGGGAAGAGA-3' |
| CCRK-genome#2-RV | 5'-CTGCCAACTATCTTCTTGCTGC-3' |
| CCRK-gRNA#1-S | 5'-CACCGGCCTTGAAGACGATGCCGT-3' |
| CCRK-gRNA#1-AS | 5'-AAACACGGCATCGTCTTCAAGGCC-3' |
| CCRK-gRNA#2-S | 5'-CACCGGCACCATACAGGAGCTCGG-3' |
| CCRK-gRNA#2-AS | 5'-AAACCCGAGCTCCTGTATGGTGCC-3' |

FW, forward; RV, reverse; S, sense; AS, anti-sense
