# Supplementary material for: CCRK/CDK20 regulates ciliary retrograde protein trafficking via interacting with BROMI/TBC1D32
Source: PLoS One. 2021 Oct 8;16(10):e0258497. doi: 10.1371/journal.pone.0258497 (PMC8500422; doi:10.1371/journal.pone.0258497)
Supplement: Attachment — Submitted filename: 210927CCRK- Response to Reviewers.docx [file pone.0258497.s009.docx]

PONE-D-21-18831R1

CCRK/CDK20 regulates ciliary retrograde protein trafficking via interacting with BROMI/TBC1D32

Dear Dr. Oliver Blacque, Academic Editor of PLOS ONE,

Thank you very much for your valuable comments and those of the reviewers on our paper submitted to PLOS ONE. According to the comments of Reviewer #3, we have made alterations in the manuscript. I am sending herewith a modified version of PONE-D-21-18831R1; see the manuscript with track changes.

I greatly appreciate your help concerning improvements of this paper. I hope that this version is now acceptable for publication in PLOS ONE.

Best regards,

Kazuhisa Nakayama, Ph.D.

**Response to the comments of Reviewer #3:**
In the abstract (“...the overaccumulation of IFT proteins at the bulged ciliary tips, which are eliminated as extracellular vesicles….”) and on Pg. 4 (“Our results showed that CCRK-KO cells accumulated IFT proteins at their bulged ciliary tips, which were eliminated as ECVs.”) the wording is still a bit strong since the conclusion is based on detection of Ift88+ punctate structures rather than live imaging of Ift88-GFP. Instead, I recommend using the wording "... appear to be eliminated as...." to soften the claim.

According to this comment, we have changed the wording to “which appear to be eliminated as extracellular vesicles,” in Abstract and “which appeared to be eliminated as ECVs.” on page 4, 3rd paragraph.

On Pg. 10 and 12, the authors write:
“The second possibility is that the abnormal phenotype of CCRK-KO cells may be attributable to the lack of phosphorylation of CCRK substrates other than ICK, including MAK, although MAK is expressed in limited tissues [31, 79].” and “The candidate substrates include MAK, a tissue-specific paralog of ICK [31], and MOK, a more divergent paralog that is also expressed in a tissue-specific manner [79], both of which have a T(D/E)Y sequence. However, in view of the fact that Mak null mice are viable [93], it is likely that ICK, which is ubiquitously expressed [79], generally contributes to the regulation of ciliary functions."

Since MAK does not appear to be expressed in RPE cells and Mak mouse mutants are viable with the only known phenotype being in photoreceptor cells, it is probably not a good idea to mention MAK as an alternative potential mediator of CCRK’s functions (so, I’d remove it). In addition, it is not clear whether MOK expressed in RPE cells. If not, I would avoid mentioning MOK as a candidate mediator as well. Please be aware that CCRK substrates/mediators don’t necessarily have to be members of the ICK/MAK/MOK family.

According to these comments, we have removed the statements containing MAK and MOK, and revised the text before and after them to make sense. (page 10, the last paragraph; and page 12, 3rd paragraph)

Considering this second possibility put forward by the authors, they should consider that different ciliary functions of CCRK could be mediated by more than one factor and that the roles of CCRK mediators may vary depending on cellular context. In human RPE cells, it could be argued that many or all of CCRK’s functions are mediated by (still unidentified) factors other than ICK, given that forced ICK-T157E expression does not rescue the IFT88 tip accumulation phenotype of Ccrk mutants. However, in mouse (NIH3T3) fibroblasts, it appears that CCRK controls ciliary length simply by phosphorylating/activating ICK and toggling of ICK between phospho states is not important. This is supported by the fact that forced expression of ICK-T157E, but not wild-type or T157A ICK, is able to rescue the ciliary length phenotype of Ccrk knockdown NIH3T3 cells (Yang et al, EMBO reports 2013).

According to this comment, we have added “although a previous study using mouse NIH3T3 cells suggested that CCRK controls ciliary length simply by phosphorylating/activating ICK [56].” in Discussion. (page 12, 2nd paragraph)
